# Development of stromal differentiation patterns in heterotypical models of artificial corneas generated by tissue engineering

**DOI:** 10.3389/fbioe.2023.1124995

**Published:** 2023-03-23

**Authors:** Cristina Blanco-Elices, Carmen Morales-Álvarez, Jesús Chato-Astrain, Carmen González-Gallardo, Paula Ávila-Fernández, Fernando Campos, Ramón Carmona, Miguel Ángel Martín-Piedra, Ingrid Garzón, Miguel Alaminos

**Affiliations:** ^1^ Tissue Engineering Group, Department of Histology, Faculty of Medicine, Universidad de Granada, Granada, Spain; ^2^ Instituto de Investigación Biosanitaria ibs.GRANADA, Granada, Spain; ^3^ GENYO, Centre for Genomics and Oncological Research: Pfizer, University of Granada, Andalusian Regional Government, PTS Granada, Granada, Spain; ^4^ Department of Biochemistry and Molecular Biology III, Faculty of Medicine, University of Granada, Granada, Spain; ^5^ Division of Ophthalmology, University Hospital San Cecilio, Granada, Spain; ^6^ Department of Cell Biology, Faculty of Sciences, University of Granada, Granada, Spain

**Keywords:** cornea, tissue engineering, Wharton’s jelly mesenchymal stem cells, stroma, basement membrane

## Abstract

**Purpose:** We carried out a histological characterization analysis of the stromal layer of human heterotypic cornea substitutes generated with extra-corneal cells to determine their putative usefulness in tissue engineering.

**Methods:** Human bioartificial corneas were generated using nanostructured fibrin-agarose biomaterials with corneal stromal cells immersed within. To generate heterotypical corneas, umbilical cord Wharton’s jelly stem cells (HWJSC) were cultured on the surface of the stromal substitutes to obtain an epithelial-like layer. These bioartificial corneas were compared with control native human corneas and with orthotypical corneas generated with human corneal epithelial cells on top of the stromal substitute. Both the corneal stroma and the basement membrane were analyzed using histological, histochemical and immunohistochemical methods in samples kept in culture and grafted *in vivo* for 12 months in the rabbit cornea.

**Results:** Our results showed that the stroma of the bioartificial corneas kept *ex vivo* showed very low levels of fibrillar and non-fibrillar components of the tissue extracellular matrix. However, *in vivo* implantation resulted in a significant increase of the contents of collagen, proteoglycans, decorin, keratocan and lumican in the corneal stroma, showing higher levels of maturation and spatial organization of these components. Heterotypical corneas grafted *in vivo* for 12 months showed significantly higher contents of collagen fibers, proteoglycans and keratocan. When the basement membrane was analyzed, we found that all corneas grafted *in vivo* showed intense PAS signal and higher contents of nidogen-1, although the levels found in human native corneas was not reached, and a rudimentary basement membrane was observed using transmission electron microscopy. At the epithelial level, HWJSC used to generate an epithelial-like layer in *ex vivo* corneas were mostly negative for p63, whereas orthotypical corneas and heterotypical corneas grafted *in vivo* were positive.

**Conclusion:** These results support the possibility of generating bioengineered artificial corneas using non-corneal HWJSC. Although heterotypical corneas were not completely biomimetic to the native human corneas, especially *ex vivo*, *in vivo* grafted corneas demonstrated to be highly biocompatible, and the animal cornea became properly differentiated at the stroma and basement membrane compartments. These findings open the door to the future clinical use of these bioartificial corneas.

## 1 Introduction

The human cornea is an optically clear organ that forms the anterior surface of the eye, and accounts for nearly two-thirds of its refractive power. Its outermost layer is the corneal epithelium, composed of non-keratinized, stratified squamous epithelial cells. Underlying the corneal epithelium, the epithelial basement membrane is a highly specialized extracellular matrix that forms a thin acellular layer playing a critical role in migration, differentiation, and maintenance of the epithelium homeostasis (Torricelli et al., 2013; Wilson et al., 2020). The corneal stroma accounts for more than 90% of the total corneal thickness, and consists of a highly organized structure with approximately 200 collagen lamellae, proteoglycans, and other components of the extracellular matrix (ECM) that are synthetized by stromal keratocytes (Chakravarti et al., 2000; Petroll and Miron-Mendoza, 2015). The inner layer of the human cornea is the corneal endothelium, which is attached to the corneal stroma by a specialized basement membrane known as the Descemet’s membrane (Torricelli and Wilson, 2014).

Corneal diseases are very frequent causes of blindness (Geoffrion et al., 2023). Although corneal transplantation is the gold-standard treatment for severe corneal diseases, the recent advances in tissue engineering offer novel therapeutic alternatives based on the use of novel bioartificial corneal substitutes (González-Andrades et al., 2017; Isaacson et al., 2018; Rico-Sánchez et al., 2019; Kostenko et al., 2022). Several models of bioengineered corneas have been described to the date, and some of them showed high levels of biomimetism with the native human cornea (Fagerholm et al., 2010, 2014; Wang et al., 2015; Xu et al., 2019; Guérin et al., 2021; Rafat et al., 2023). Most bioengineered cornea substitutes are based on a combination of different types of biomaterials and human corneal cells obtained from sclerocorneal limbus biopsies. Among the few advanced therapy medicinal products (ATMP) that have been granted marketing authorization in Europe, Holoclar contains *ex vivo* expanded autologous human corneal epithelial cells cultured on a fibrin scaffold (Pellegrini et al., 2018). Although these products have shown positive results in patients, the fabrication method requires obtaining a biopsy from a healthy eye, and this surgical procedure is not free from the possibility of complications and side effects (Shortt et al., 2011). For this reason, heterotypical models of bioartificial human corneas have been developed using alternative non-corneal cells such as the human Wharton’s jelly stem cells obtained from the umbilical cord (HWJSC) that are not based on limbal biopsies (Garzón et al., 2014; Garzon et al., 2020). Although these heterotypical corneas were previously evaluated at the epithelial level and demonstrated to have differentiation capability during the 3 weeks required for these tissues to be biofabricated (Garzón et al., 2020), the characteristics of the bioartificial stroma and the basement lamina of these tissues have not been determined to the date.

In the present work, we have developed and characterized a heterotypic model of the human cornea both *ex vivo* and *in vivo* to determine the main features of the stroma and basement lamina of these bioartificial corneas.

## 2 Materials and methods

### 2.1 Primary cell cultures

Primary cell cultures of human corneal epithelial and stromal cells were established from samples of human limbal scleral rings as previously reported (Garzón et al., 2020). Epithelial cells were cultured with epithelial medium (EM) consisting of a mixture of Ham-F12 (150 mL), Dulbecco’s modified Eagle’s medium (DMEM) (300 mL) and fetal bovine serum (FBS) (50 mL), supplemented with 1% antibiotics/antimycotics, adenine (24 μg/mL), insulin (5 μg/mL), triiodothyronine (1.3 ng/mL), hydrocortisone (0.4 μg/mL) and epidermal growth factor (EGF) (10 mg/mL). Corneal stromal cells were cultured with SM stromal medium consisting of DMEM with 10% FBS and 1% antibiotics/antimycotics. All these products were purchased from Merck (Darmstadt, Germany). Cultures of human Wharton’s Jelly mesenchymal stem cells (HWJSC) were purchased from Merck. HWJSC were cultured with Amniomax™ culture medium (Gibco-Thermo Fisher Scientific, Waltham, MA). In all cases, cells were cultured at 37°C in a humidified incubator with 5.0% CO_2_ using standard cell culture conditions. The culture medium was renewed every 3 days.

This research was performed in accordance with guidelines and regulations of the Association for Research in Vision and Ophthalmology (ARVO) for the use of animals in ophthalmic and vision research. This project was approved by the local Human Research and Ethics Committee of the province of Granada (PEIBA), numbers 1915-N-20 and 2224-N-20, and all tissue donors provided their informed consent to participate in the project.

### 2.2 Generation of orthotypical and heterotypical bioengineered human corneas

A biological substitute of the human corneal stroma was first generated using fibrin-agarose biomaterials with stromal cells immersed within. To generate 1 mL of stroma substitute, we mixed 760 µL of human plasma, 75 µL of DMEM containing 100,000 cultured human stromal cells, 15 µL of tranexamic acid (Amchafibrin, Fides-Ecofarma, Valencia, Spain), 50 µL of a 2% solution of type VII agarose (Merck) melted in PBS (Merck), and 100 µL of 1% CaCl_2_ (Merck). This mixture was aliquoted on 6 well plates with 24 mm Transwell porous inserts (Sarstedt, Nümbrecht, Germany) and incubated at 37°C until complete jellification of the biomaterial.

Then, a stratified epithelial layer was generated on top of the stromal substitute, using cultured corneal epithelial cells for the orthotypical bioartificial corneas (OAC) or HWJSC for the heterotypical bioartificial corneas (HAC). Cultured cells were trypsinized, and 1 × 10^6^ cells were subcultured on top of each stroma substitute. Both the OAC and HAC were kept for 21 days in a cell culture incubator at 37°C, and the culture medium was renewed every 3 days. Stratification and differentiation of the epithelial layer was promoted by using the air-liquid culture technique from day 14 to day 21, as previously reported (Reichl and Müller-Goymann, 2003).

### 2.3 *In vivo* evaluation of orthotypical and heterotypical bioengineered human corneas

To characterize *in vivo* both models of bioengineered artificial cornea (OAC and HAC), bioengineered corneas were grafted on the eye surface of 20 New Zealand laboratory rabbits using the partial-thickness corneal transplant technique (DALK). All animals were 9-month-old adult males. Rabbits were deeply anesthetized by intramuscular injection of xylazine (5 mg/kg) (Calier S.A., Barcelona, Spain) and ketamine (25 mg/kg) (Merial labs, Barcelona, Spain). Then, a circular incision 6 mm in diameter and 200 µm deep was made in the cornea of the right eye of each animal using a manual keratotome, and the most external layers of the cornea were excised. First, an air bubble was injected into the cornea using a needle, and the external layers of the cornea were removed using a ophthalmological scalpel, and the surface of the cornea was carefully cleaned from any rests of remaining tissue using microsurgical forceps. Then, OAC and HAC artificial corneas were grafted on the corneal stroma and sutured with 10/0 nylon stitches (B. Braun, Melsungen, Germany). Local antibiotics and anesthetic drops were instilled after surgery (Alcon, Geneva, Switzerland). The left eye was not operated. Animals were followed-up for up to 12 months. A macroscopic evaluation was carried out on each animal, and slit lamp and OCT images were obtained from the right and left eyes after 3 and 12 months of the implant. Corneal opacity was graded according to the classification established by García and cols (Garcia et al., 2021). as mild (grade I), moderate (grade II) and dense (grade III).

Animal experimentation was approved by the regional research and ethics committee for animal experimentation (CEEA), ref. numbers 16/12/2020/147 and 25/06/2018/099.

### 2.4 Histological analyses

For histological analysis, artificial cornea models and control human corneas were fixed in 4% formaldehyde and embedded in paraffin (both, from PanReac, Barcelona, Spain) following standard protocols. Histological sections with a thickness of 5 µm were obtained, deparaffinized with xylene (PanReac), cleared in ethanol (PanReac) and rehydrated in water. For the analysis of general structure, tissue sections were stained with Masson’s trichrome staining method. In brief, samples were incubated for 15 min in solution A (0.5 mL of acid fuchsin, 0.5 mL of glacial acetic acid and 99 mL of distilled water), 10 min in solution B (1 g of phosphomolybdic acid and 100 mL of distilled water) and 5 min in solution C (2 g of light green dye, 1 mL of glacial acetic acid and distilled water up to 100 mL). Samples were then washed in distilled water, dehydrated in alcohol and coverslipped. All these reagents were purchased from PanReac.

Transmission electron microscopy (TEM) ultrastructural analyses were carried out using routine methods. In brief, tissues were fixed for 8 h in 2.5% glutaraldehyde (PanReac), washed three times in cacodylate buffer (PanReac) and postfixed for 90 min in 1% osmium tetroxide (Merck). After fixation, samples were dehydrated with increasing acetone series (PanReac), embedded in epoxy resin (Merck), and cut in ultrathin sections of 60 nm using a Leica ultracut UCT ultramicrotome (Leica Mycrosystems GmbH, Wetzlar, Germany). Sections were stained with aqueous uranyl acetate and lead citrate (both, from Merck) and evaluated with an EM902 transmission electron microscope operating at 80 kV (Carl Zeiss Meditec, Inc., Oberkochen, Germany).

### 2.5 Histochemical analysis of ECM components in OAC and HAC samples

Evaluation of relevant fibrillar and non-fibrillar components of the tissue ECM was carried out by using specific histochemical methods, as previously described (Vela-Romera et al., 2019). To evaluate the presence of collagen fibers in CTR and bioengineered corneas, tissue sections were stained with picrosirius red histochemistry (PSR). In brief, samples were incubated in sirius red F3B reagent for 30 min, washed in water and counterstained with Harris hematoxylin for 5 min. Samples stained with PSR were evaluated with polarized light to reveal the presence of mature collagen fibers. To identify reticular fibers, tissue sections were stained using the reticulin metal reduction method of Gomori (RET). In this case, samples were incubated in 1% potassium permanganate, followed by 2% sodium metabisulphite solution and sensibilization with 2% iron alum. After that, samples were incubated in ammoniacal silver and in 20% formaldehyde. Differentiation was performed with 2% gold chloride and 2% thiosulphate. Detection of elastic fibers was carried out using the histochemical method of Verhoeff (VER). Samples were incubated in the Verhoeff staining solution for 10 min, with differentiation in 2% ferric chloride for 15 s. Identification of tissue proteoglycans was performed by Alcian blue histochemistry (AB) was used to identify acid proteoglycans (sulfated and carboxylated) and mucosubstances. Tissue sections were incubated in alcian blue working solution for 30 min and counterstained with nuclear fast red for 1 min. Finally, the analysis of saccharide groups containing 1,2 glycol groups typically associated with glycoproteins, neutral gel-forming mucins and polysaccharides, including those found in the basement membrane was carried out by periodic acid–Schiff staining (PAS). Tissues were incubated in 0.5% periodic acid solution for 5 min, followed by incubation in Schiff reagent for 15 min and slight counterstaining with Harris’s hematoxylin for 20 s. All these reagents were purchased from PanReac. In all cases, slides were dehydrated and coverslipped, and histological images were obtained using an Eclipse 90i light microscope (Nikon, Tokyo, Japan) or a Pannoramic Desk DW II histological scanner (3DHISTECH, Budapest, Hungary).

### 2.6 Immunohistochemical analysis of ECM components in OAC and HAC samples

To evaluate the corneal epithelial cell phenotype, we used immunohistochemistry for p63, a marker of immature proliferating epithelial cells. To quantify the presence of three specific small leucine-rich proteoglycans playing an important role in the corneal stroma, we used decorin, keratocan and lumican immunohistochemistry. Finally, analysis of the presence of nidogen-1 at the basement membrane of control and bioengineered corneas was performed by immunohistochemistry using specific antibodies anti-nidogen1. In all cases, deparaffinized tissue sections were subjected to antigen retrieval with citrate (PanReac) at 98°C for 20 min, followed by endogenous peroxidase blocking with 3% of H_2_O_2_ in methanol (Merck) and non-specific antibody binding was prevented with casein and normal horse serum (Vector Laboratories, Burlingame, CA). Samples were then incubated overnight with one of the following primary antibodies at 4°C in a humid chamber: anti-p63 (Master Diagnostica, Granada, Spain, MAD-000479QD, monoclonal, dilution 1:500), anti-decorin (Rf&D Systems, Minneapolis, MN, AF143-SP, polyclonal, dilution 1:500), anti-keratocan (Novus Biologicals, Littleton, CO, NBP1-84425, polyclonal, dilution 1:50), anti-lumican (R&D Systems, AF2846, polyclonal, dilution 1:100), anti-nidogen-1 (R&D Systems, AF2570, polyclonal, dilution 1:50). Samples were then incubated in HRP-conjugated secondary antibodies (Vector Laboratories) for 1 h, and the immunohistochemical staining signal was revealed using diaminobenzidine (DAB) (Vector Laboratories). Immunostaining was contrasted with Harris’s hematoxylin (PanReac), and slides were dehydrated and coverslipped. Images were obtained using a Pannoramic Desk DW II histological scanner (3DHISTECH). To identify human cells, tissue sections were dewaxed, treated with EDTA (PanReac) pH = 8 at 90°C for 20 min for antigen retrieval, and preincubation was performed with casein and normal horse serum (Vector). Tissues were then incubated with anti-human mitochondria antibodies (Merck, monoclonal, dilution 1:80) overnight at 4°C, followed by three washes in PBS and incubation in a FITC-conjugated secondary antibodies (Vector Laboratories). Slides were then coverslipped using fluorescence mounting medium (Vector Laboratories) and histological images were obtained using a Nikon Eclipse i90 fluorescence microscope.

### 2.7 Quantification and statistical analysis

Once each histological image was obtained from each sample, we carried out a quantitative analysis of the presence of relevant ECM components of the corneal stroma and basement membrane. Images were analyzed using the software ImageJ (National Institutes of Health, Bethesda, MD) as previously described (Fermin et al., 1992; Vela-Romera et al., 2019). For the ECM components of the corneal stroma, the relative amount of each component was quantified in each sample by using the area fraction method of ImageJ. For each image, we first selected the specific color channel corresponding to each staining method using the “split channels” tool of the software, using technical control samples as a reference. Then, images were converted to binary (black and white) images, and a square with an area of 50 µm × 50 µm was drawn in the stromal layer. Then, the percentage of each analyzed tissue that was occupied by each specific ECM component was quantified using the area fraction tool of the program. For the PAS and nidogenin-1 staining signal, images converted to binary were analyzed using the single-point tool of the software at the basement membrane. By using this method, the intensity corresponding to each analysis method was quantified in the basement membrane of each sample. All values were quantified in intensity units (i.u.) of the ImageJ software. For p63, we quantified the total number of epithelial cells and the number of cells showing positive immunohistochemical staining for this marker in each type of sample, and the percentage of p63-positive cells was calculated. Eight different samples (n = 8) were analyzed for each staining method and each type of sample (CTR, OAC and HAC corresponding to each analysis time).

Comparisons of the quantitative results were carried out using Mann-Whitney U tests, since our initial analysis using the test of Shapiro-Wilk demonstrated that most variables were not parametric and did not fit a normal distribution. For each component of the corneal stroma and basement membrane, results obtained for CTR native human corneas were compared with values corresponding to each study group (OAC and HAC kept *ex vivo* for 7, 14 and 21 days and OAC and HAC grafted *in vivo* for 3 and 12 months). In addition, each time point was compared with the next time point for OAC and HAC (for example, OAC cultured *ex vivo* for 7 days were compared with OAC cultured es *vivo* for 14 days), and OAC corresponding to a specific time point were compared with HAC of the same time point (for example, OAC cultured *ex vivo* for 7 days were compared with HAC cultured *ex vivo* for 7 days). A Bonferroni-adjusted *p*-value ≤ 0.001 was set as statistically significant to correct for multiple testing.

## 3 Results

### 3.1 Ophthalmologic analysis

**FIGURE 1:**
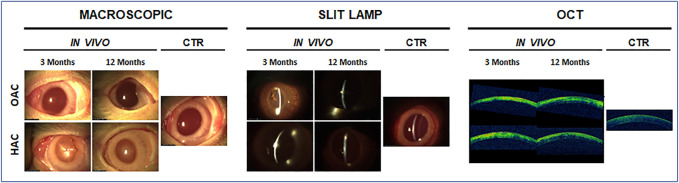
Ophthalmologic analysis of rabbit eyes grafted with the orthotypical (OAC) and heterotypical (HAC) artificial corneas and control eyes (CTR). Macroscopic photographs, slit lamp observations and optical coherence tomography images (OCT) are shown for each type of sample after 3 and 12 months of *in vivo* follow-up.

The ophthalmologic analysis of the rabbit eyes grafted with the bioartificial corneas confirmed our previous reports showing the biocompatibility of OAC and HAC tissues (Garzón et al., 2020). As shown in Figure 1, the implant of both types of bioartificial corneas was safe for the host animal, and no infection, rejection or tumorigenesis were observed after 3 and 12 months. Although some inflammatory signs were detected after 3 months, results at 12 months were compatible with a normal process of biointegration of the bioartificial corneas in the host tissue. These results were confirmed by macroscopic examination, slip lamp analysis and OCT scanning. In fact, our ophthalmologic analysis revealed that the opacity of bioartificial corneas grafted in animals tended to decrease with time. As shown in Figure 1, OAC showed no opacity after 3 and 12 months of follow-up, being comparable to CTR native corneas. However, HAC showed mild (grade I) opacity at 3 months, which disappeared at 12 months. Analysis of these corneas using slit lamp and OCT evaluation confirmed that the density of the corneal opacity was very low, especially in OAC, and tended to disappear after 12 months of the implant.

### 3.2 Histological analysis of OAC and HAC

**FIGURE 2:**
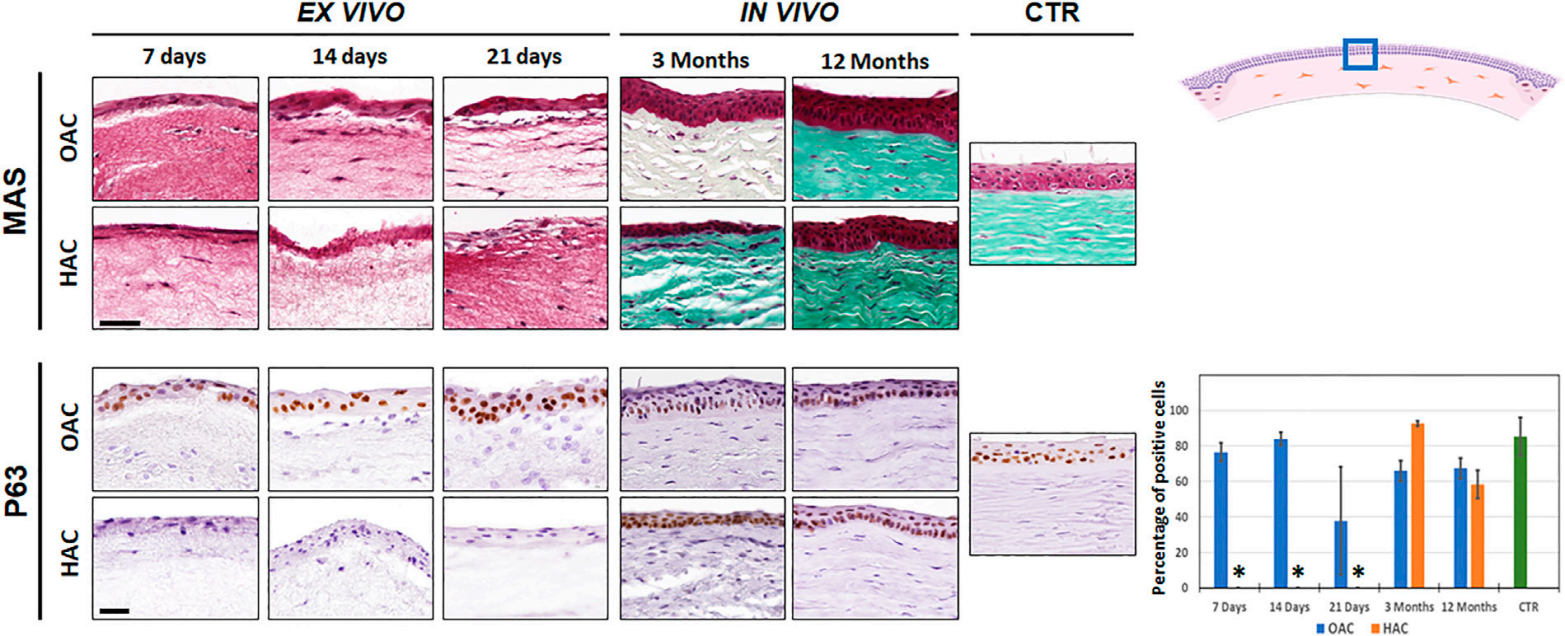
Histological evaluation of orthotypical (OAC) and heterotypical (HAC) artificial corneas and controls. The top panel corresponds to the analysis using Masson’s trichrome staining (MAS), whereas the lower panel shows the immunohistochemical analysis of p63. In both cases, the area in which the *in vivo* histological images were obtained is labeled with a blue square in the illustration representing the rabbit cornea shown at the upper right corner. Bioartificial corneas kept *ex vivo* for 7, 14 and 21 days, and corneas grafted *in vivo* for 3 and 12 months are shown. Controls (CTR) correspond to native, normal human corneas. Scale bars: 50 µm (applicable to all images). The histogram corresponding to the p63 panel shows the results of the quantitative analysis of the percentage of positive cells in each group. Asterisks represent statistically significant differences as compared to CTR corneas.

Histological characterization of OAC and HAC kept *ex vivo* using Masson’s trichrome staining (MAS) revealed the presence of a stroma substitute containing stromal cells immersed within a randomly-organized fibrillar mesh, and a stratified epithelial cell layer on top (Figure 2). Once grafted *in vivo*, we found a dense corneal stroma consisting of numerous lamellae of well-organized fibers with abundant stromal cells allocated among the lamellae. At the epithelial level, *in vivo* samples showed a well-differentiated epithelium with numerous cell strata, especially at 12 months. Both the stroma and the epithelial layers resembled the control native corneas, after 12 months of follow-up in OAC and HAC. To characterize the epithelial layer of each type of cornea, we analyzed the expression of p63. Results showed a high percentage of positive cells in OAC samples kept *ex vivo*, but HAC were negative for this marker. *In vivo*, corneas implanted with either OAC or HAC, subsequently regenerated an epithelium expressing p63, as it was the case of the control corneas (Figure 2). Statistically significant differences were found between CTR corneas and HAC kept *ex vivo*, but not with the rest of samples analyzed here. In addition, analysis of human cells using specific antibodies showed positive immunofluorescence signal in the human control cornea and HAC samples, although the signal tended to be stronger in samples kept *ex vivo* (Supplementary Figure S1).

### 3.3 Analysis of fibrillar ECM components in OAC and HAC

**FIGURE 3:**
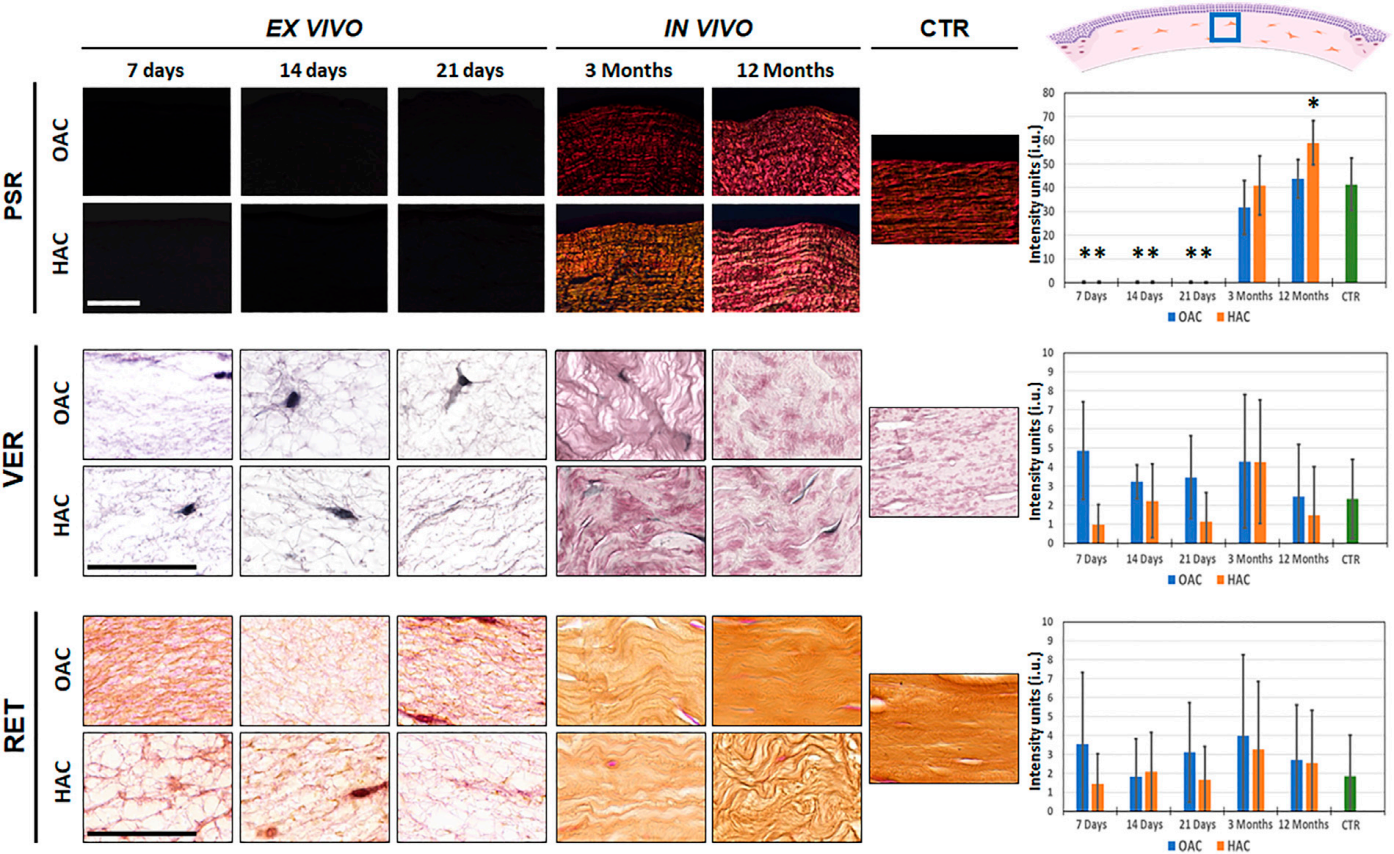
Identification of fibrillar components of the corneal stroma of orthotypical (OAC) and heterotypical (HAC) artificial corneas and controls. PSR: picrosirius red histochemistry for collagen fibers detection; VER: Verhoeff histochemistry for elastic fibers; RET: Gomori’s reticulin histochemistry for reticular fibers. The area in which the *in vivo* histological images were obtained is labeled with a blue square in the illustration representing the rabbit cornea shown at the upper right corner. Bioartificial corneas kept *ex vivo* for 7, 14 and 21 days, and corneas grafted *in vivo* for 3 and 12 months are shown. Controls (CTR) correspond to native, normal human corneas. Scale bars: 50 µm (applicable to all images). Histograms to the right correspond to the quantitative analysis of signal intensity quantified in intensity units (i.u.) of the ImageJ software. Asterisks represent statistically significant differences as compared to CTR corneas.

When the presence of collagen fibers was evaluated in bioartificial corneas using picrosirius red staining, we found that tissues kept *ex vivo* were mostly devoid of these fibers. However, *in vivo* grafting was associated with a significant increase of the synthesis of collagen fibers, with positive PSR signal in OAC and HAC samples grafted for 3 and 12 months in laboratory animals. The signal found in OAC samples grafted *in vivo* and HAC corresponding to 3 months of *in vivo* follow-up was comparable to CTR corneas (*p* > 0.05). However, analysis of HAC after 12 months of follow-up revealed a significant increase of collagen fibers intensity as compared to CTR (*p* = 0.001) (Figure 3 and Supplementary Tables S1, S2). On the other hand, the analysis of elastic and reticular fibers as determined by Verhoeff and reticulin staining revealed that all bioartificial corneas, including *ex vivo* and *in vivo* samples, and CTR corneas showed very low staining signal, suggesting that both types of samples contained very few of these types of fibers, with non-significant differences among samples (Figure 3 and Supplementary Tables S1, S2).

### 3.4 Analysis of non-fibrillar ECM components in OAC and HAC

**FIGURE 4:**
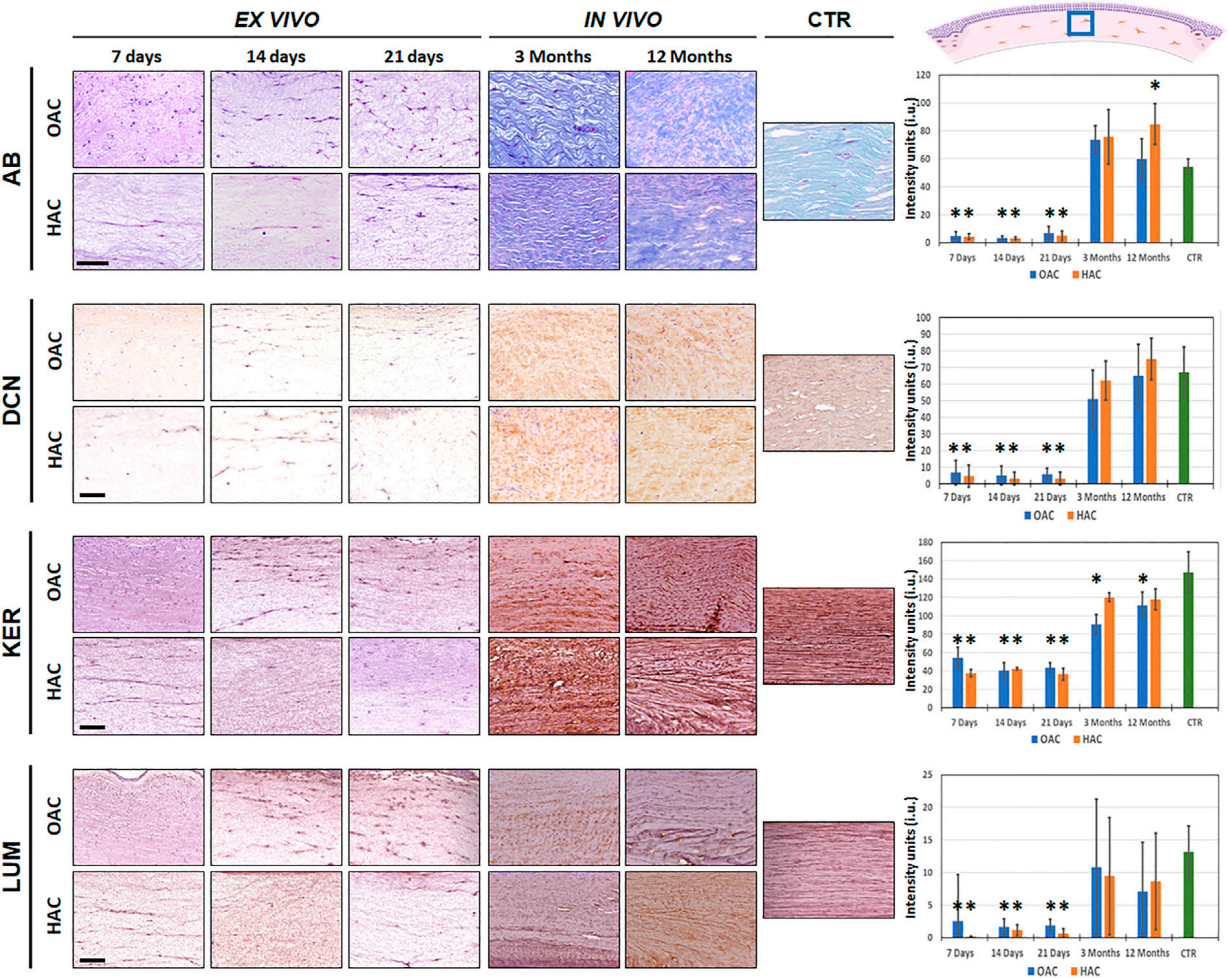
Identification of non-fibrillar components of the corneal stroma of orthotypical (OAC) and heterotypical (HAC) artificial corneas and controls. AB: alcian histochemistry for global proteoglycan detection; DCN, KER and LUM: immunohistochemical detection of the proteoglycans decorin, keratocan and lumican, respectively. The area in which the *in vivo* histological images were obtained is labeled with a blue square in the illustration representing the rabbit cornea shown at the upper right corner. Bioartificial corneas kept *ex vivo* for 7, 14 and 21 days, and corneas grafted *in vivo* for 3 and 12 months are shown. Controls (CTR) correspond to native, normal human corneas. Scale bars: 50 µm (applicable to all images). Histograms to the right correspond to the quantitative analysis of signal intensity quantified in intensity units (i.u.) of the ImageJ software. Asterisks represent statistically significant differences as compared to CTR corneas.

First, we quantified the presence of proteoglycans identified by alcian blue staining (Figure 4 and Supplementary Tables S1, S2). Results revealed that these non-fibrillar components of the corneal stroma ECM were very scarce in bioartificial corneas kept *ex vivo*, with no differences between culture times and between OAC and HAC (*p* > 0.05). However, *in vivo* implantation was associated to a significant increase in the amount of proteoglycans, and the staining intensity of OAC and HAC corresponding to 3 months of *in vivo* follow-up, along with OAC at 12 months, was comparable to CTR samples. Interestingly, HAC implanted *in vivo* for 12 months showed significantly higher concentrations of proteoglycans than CTR native corneas (*p* = 0.0006).

In the second place, we carried out a quantitative analysis to determine the presence of three important small leucine-rich proteoglycans in the corneal stroma (Figure 4 and Supplementary Tables S1, S2). Our results showed that decorin and lumican were detectable at very low concentrations in bioartificial corneas kept *ex vivo*, without differences between OAC and HAC or among culture times. However, we found that *in vivo* implantation resulted in a significant increase of both proteoglycans, and all *in vivo* samples were similar to CTR corneas (*p* > 0.05). Nevertheless, quantification of keratocan showed that all samples contained certain amounts of this proteoglycan, although the levels found in *ex vivo* samples were significantly lower than CTR (*p* < 0.001). When corneas were grafted on laboratory rabbits, we found a significant increase of keratocan staining signal, although this level was comparable to CTR only in the case of HAC samples (at 3 and 12 months), with statistically significant differences between OAC and CTR after 3 and 12 months of follow-up (*p* = 0.0001 and *p* = 0.0006, respectively).

### 3.5 Analysis of basement membrane components in OAC and HAC

**FIGURE 5:**
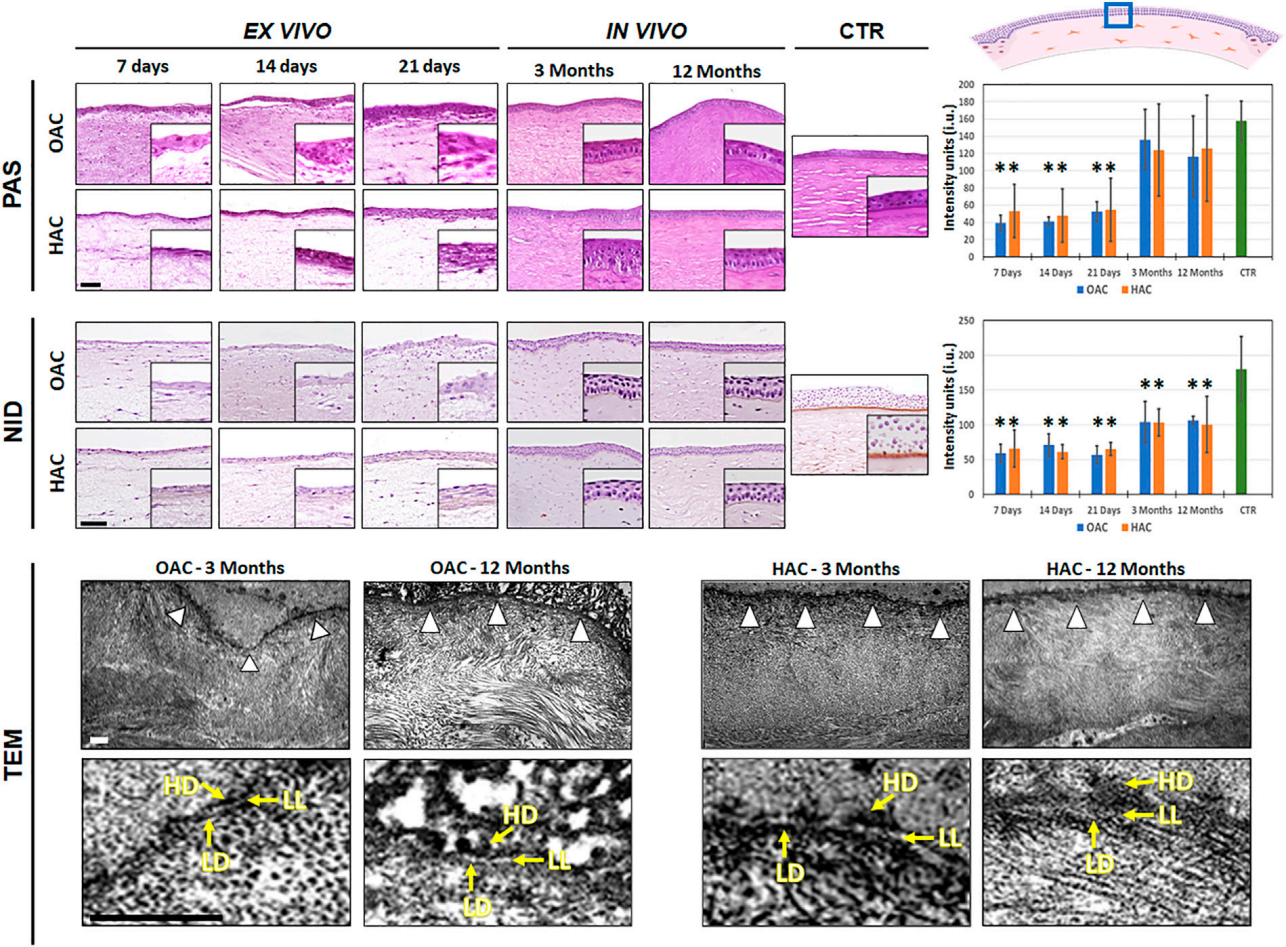
Analysis of the basement membrane in orthotypical (OAC) and heterotypical (HAC) artificial corneas and controls. PAS: periodic acid of Schiff histochemistry for glycosaminoglycans of the basement membrane; NID: nidogen-1 immunohistochemistry. In both cases, the area in which the *in vivo* histological images were obtained is labeled with a blue square in the illustration representing the rabbit cornea shown at the upper right corner. TEM: transmission electron microscopy with white arrowheads pointing to the basement membrane and yellow arrows highlighting the presence of the *lamina lucida* (LL), *lamina densa* (LD) and hemidesmosomes (HD). Bioartificial corneas kept *ex vivo* for 7, 14 and 21 days, and corneas grafted *in vivo* for 3 and 12 months are shown for the histochemistry and immunohistochemistry analyses. Controls (CTR) correspond to native, normal human corneas. Scale bars: 50 µm for the histochemistry and immunohistochemistry and 1 µm for the TEM analysis (applicable to all images). Histograms to the right correspond to the quantitative analysis of PAS and NID signal intensity quantified in intensity units (i.u.) of the ImageJ software.

Apart from the study of proteoglycans, we assessed the presence of specific components of the basement membrane using PAS and NID staining (Figure 5 and Supplementary Tables S1, S2). For PAS histochemistry, our results revealed that glycoproteins were detectable at low levels in all *ex vivo* samples, with statistically significant differences with CTR (*p* < 0.001). In contrast, OAC and HAC grafted *in vivo* showed a significant increase of these components, and the PAS intensity found at the basement membrane of all bioartificial corneas implanted on laboratory animals were comparable to CTR corneas (*p* > 0.05). When the presence of nidogen-1 was assessed by immunohistochemistry, we also found that the amounts of this protein at the basement membrane of corneas kept *ex vivo* was significantly lower as compared to CTR human corneas, and that *in vivo* grafting was able to increase the presence of this component of the basement membrane. However, the amounts of nidogen-1 were significantly lower than CTR in all samples, including those grafted *in vivo* (Figure 5 and Supplementary Tables S1, S2).

The histological characterization of the basement membrane of OAC and HAC using TEM revealed that this structure was not detectable in samples kept *ex vivo*, and was identified only in bioartificial corneas grafted on laboratory animals. As shown in Figure 5, an electrodense linear structure was found between the stromal and the epithelial layers of OAC and HAC at 3 and 12 months. As expected, this structure consisted of an electron-lucid layer corresponding to the *lamina lucida* and an electron-dense layer corresponding to the *lamina densa*. Interestingly, hemidesmosomes were found at the basal cell membrane of the epithelial cells contacting the basement membrane, in OAC and HAC corresponding to 3 and 12 months of *in vivo* follow-up.

## 4 Discussion

One of the main challenges in the construction of bioartificial tissues and organs by tissue engineering is obtaining viable cells allowing the generation of the artificial tissue in a minimum of time. Although corneal stem cells previously demonstrated their capability to generate a well-differentiated corneal epithelium in laboratory (Guérin et al., 2021), the limitations related to the need of obtaining limbal biopsies and the lack of functional limbal cells in patients affected by limbal stem cell deficiency, make necessary the search for alternative extra-corneal cells able to reproduce the corneal epithelium (Garzón et al., 2014). In fact, several works have been focused on the generation of an efficient corneal epithelium using different strategies that include the use of corneal and non-corneal cell sources (Klenkler et al., 2010; Ma et al., 2011; Nosrati et al., 2021).

In this regard, our research group previously demonstrated the potential usefulness of HWJSC to generate epithelial-like cell layers in several models of bioengineered human tissues, including the urothelial mucosa (Garzón et al., 2021), the skin epidermis (Martin-Piedra et al., 2019), oral mucosa epithelium (Alfonso-Rodríguez et al., 2015) and also the corneal epithelium (Garzón et al., 2014). In the case of the human cornea, the use of HWJSC were able to stratify and differentiate very efficiently on a bioartificial corneal stroma, especially when the bioengineered corneas were grafted *in vivo* (Garzón et al., 2020). In a previous work, we demonstrated that these non-corneal cells were able to generate a well-differentiated corneal epithelium expressing typical markers that include several cytokeratins, intercellular junctions and crystallins (Garzón et al., 2020).

In the present work, we have characterized the stromal layer and the basement membrane of these bioartificial human corneas to determine its homology and degree of biomimicry with the native human cornea. This characterization will contribute to determine their potential clinical utility, since a proper characterization of the whole organ is a crucial requirement for organs intended for future clinical use (Rico-Sánchez et al., 2019). In general, our results suggest that HAC generated with HWJSC could be partially analogue to the human cornea, and both the HAC and the OAC models developed in this work contained an epithelial and a connective layer reaching similar differentiation patterns to native control corneas, and confirmed that these bioartificial tissues were biocompatible when grafted *in vivo*, as previously reported (Garzón et al., 2020). The fact that bioartificial corneas tended to show progressive levels of transparency and the initial mild opacity found in some corneas disappeared at 12 months of follow-up supports the putative future usefulness of these artificial tissues. Although it is well-known that the host sclerocorneal limbus could be able to replace most epithelial cells by host cells as a result of the physiological epithelial turnover, our analysis of the tissues grafted *in vivo* using specific anti-human cell antibodies showed some positive immunostaining signal. Though some of the grafted cells might remain *in situ* at the host cornea, it could also be possible that positivity to anti-human mitochondria could be explained by transference of human mitochondria from the grafted corneas to the host epithelium. In fact, previous reports demonstrated that intercellular mitochondrial transfer may occur among different types of cells, and transferring could be mediated by tunnelling nanotubes, extracellular vesicles, and gap junction channels (Liu et al., 2022).

When the epithelial layer of our cornea substitutes was analyzed, we found that the HWJSC used to replace the epithelial layer of our bioartificial corneas were able to partially mature and differentiate on top of the corneal stroma kept *ex vivo*, showing few signs of epithelial differentiation. After *in vivo* implantation, these corneas were able to integrate in the host animal after the established follow-up time. Interestingly, the epithelial-like cell layer found in the HAC model kept *ex vivo* was not able to express p63, a regulator of epithelial differentiation and morphogenesis (Chee et al., 2006; Novelli et al., 2022), confirming the non-epithelial phenotype of these cells. However, the use of corneal epithelial cells in OAC models was indeed associated with the positive expression of this marker, as it is the case of the human native cornea. p63 proteins are known to play crucial roles in regulating cell cycle in normal and tumoral cells, and is considered to be a marker of several types of immature proliferating epithelial cells (Nekulova et al., 2013). These results confirm the idea that the epithelial-like cell layer found in HAC was poorly differentiated while kept *ex vivo* and become integrated, resembling the native cornea, once grafted on the eye surface of laboratory animals. Although longer times of *ex vivo* culture should be analyzed, these results are in agreement with previous reports suggesting that bioartificial human tissues generated by tissue engineering are partially differentiated while kept in culture and tend to increase differentiation once grafted *in vivo* by means of several paracrine and juxtracrine factors synthetized by the host animal and by epithelial-mesenchymal interactions (Garzón et al., 2020). In the case of HAC, it is likely that these factors can contribute to the normal physiology and homeostasis of the grafted cornea, and *in vivo* samples are able to express p63, once implanted *in vivo*. As the role of p63 has been previously described as essential in controlling epithelial proliferation, differentiation and development (Novelli et al., 2022), these results are in agreement with previous reports suggesting that mesenchymal stem cells could have the potential to improve tissue regeneration, and may have *in vivo* differentiation potential (Garzón et al., 2020).

One of the most important layers of the human cornea is the stroma, which represents more than 90% of the total corneal thickness. In normal native corneas, this layer consists of a dense network of properly-oriented collagen fibers and proteoglycans, in which corneal stromal cells reside (Espana and Birk, 2020). Analysis of the structure and composition of the corneal stroma is crucial, since these parameters play a key role in controlling its physical and optical properties, including corneal transparency (Torricelli et al., 2013; Torricelli and Wilson, 2014). In agreement with the results found for the epithelial layer, we found that the corneal stroma ECM was poorly differentiated in corneas kept *ex vivo* for 3 weeks, and the *in vivo* environment was able to induce this layer to differentiate and mature, especially after long *in vivo* follow-up times. Regarding the fibrillar components of the corneal stroma, we found that artificial corneas kept *ex vivo* consisted in human stromal cells immersed in a fibrin-agarose biomaterial whose fibers were not oriented, and we did not find the presence of collagen, elastic or reticular fibers. As expected, this ECM was significantly remodeled after *in vivo* implantation, and we found very abundant and properly-oriented collagen fibers and a total absence of elastic and reticular fibers in all samples grafted *in vivo* resembling the native human cornea. Again, the *in vivo* microenvironment was likely responsible for the terminal differentiation of the stromal layer, as it is well-known that cells and tissues kept in culture are typically found in a poorly differentiated state (Lee et al., 2015). An interesting finding is that the stroma of HAC contained more collagen fibers than control human corneas after 12 months of follow-up. This finding could be related with the intrinsic nature of HWJSC, which are known to be metabolically active cells able to synthetize large amounts of ECM components that can be harvested and used as natural biomaterials (Dan et al., 2017; Garzon et al., 2020). These results are in agreement with our previous analysis of the optical properties of the artificial corneas showing that the light transmittance properties of tissues grafted *in vivo* is significantly higher than those of corneas kept *ex vivo*, as it is well known that a proper organization of the stromal fibers is crucial for corneal transparency (Garzón et al., 2020).

Besides the fibrillar components of the corneal stroma, we have also evaluated the presence of non-fibrillar components of the stroma ECM. Previous reports described that cornea ECM maturation is directly linked to a homogenous distribution of collagen fibrils surrounded by proteoglycans, especially decorin, keratocan and lumican, which are typically formed by tandem repeats of approximately 25 aminoacids (Torricelli and Wilson, 2014). In concordance with the results found for the fibrillar components, our analysis showed that bioartificial corneas kept *ex vivo* were devoid of most of these components at all study times, suggesting that the stroma of corneas kept in culture were poorly differentiated. However, *in vivo* grafting was associated with an increment in the synthesis of proteoglycans, including decorin, keratocan and lumican, reaching the levels found in control corneas in corneas grafted *in vivo*, with the exception of keratocan in OAC samples. Strikingly, HAC grafted for 12 months expressed higher amounts of proteoglycans than control corneas, and similar amounts of keratocan than control corneas. Again, the metabolically active nature of these cells and their capability to synthetize fibrillar and non-fibrillar components of the human ECM could explain these results (Dan et al., 2017; Garzon et al., 2020). In fact, it has been reported that the Wharton’s jelly is a mucosal embryonic-like connective tissue whose cells are committed to synthesize large amounts of proteoglycans in the umbilical cord (Gogiel et al., 2003). Again, the presence of these non-fibrillar components of the ECM in corneas grafted *in vivo* could contribute to explain our previous findings demonstrating that *in vivo* corneas show higher levels of transparence than *ex vivo* samples (Garzón et al., 2020). In addition, the fact that *in vivo* HAC contained higher amounts of keratocan than OAC, especially after 3 months of *in vivo* implantation, could explain why HAC showed better optical properties than OAC at this specific time (Garzón et al., 2020).

In order to complete our histological characterization of the bioartificial cornea models generated in this work, we also evaluated the basement membrane in these artificial tissues. The basement membrane is a highly specialized acellular extracellular matrix able to control cell physiology, tissue regeneration and homeostasis, and its presence is fundamental for the development and adhesion of the epithelial layer (Wilson et al., 2020). At the ultrastructural level, the basement membrane consists of a lamina lucida and a lamina densa, which can be visualized using TEM methods (Torricelli et al., 2013). In the case of the human cornea, this structure contains abundant glycosaminoglycans that can be identified by PAS histochemistry (Folberg et al., 2000) and nidogen-1, among other components (Kruegel and Miosge, 2010). In our work, we found that the basement membrane was detectable only in bioengineered corneas grafted *in vivo*, with both the OAC and HAC showing a PAS-positive basement membrane underlying the epithelial layer, and the staining intensity of these corneas implanted *in vivo* was similar to control corneas. These results are in agreement with the visualization of a structure compatible with a basement membrane when *in vivo* corneas were analyzed by TEM, in which the typical lamina lucida and lamina densa structures were detectable. As in the case of the epithelial and stromal layers, these results support the idea that the *in vivo* environment is able to induce the differentiation of a basement membrane mediated by epithelial-stromal interactions. However, the fact that *in vivo* samples showed significantly lower amounts of nidogen-1 than control corneas could also suggest that differentiation could not be complete in bioartificial corneas grafted *in vivo* for 3 and 12 months of follow-up.

As it is well known that the regeneration capability of the cornea is limited, and full regeneration can take up to 5 years after surgery (Yam et al., 2017), we may hypothesize that a full differentiation and synthesis of nidogen-1 by the basement membrane and other ECM components could require longer periods of time. In the present work, we analyzed the bioartificial corneas kept *ex vivo* for 3 weeks in order to reproduce the actual situation of HAC generated as ATMP for clinical use in a clinical trial (González-Andrades et al., 2017). However, it is very likely that longer periods of *ex vivo* culture be associated with the synthesis of significant amounts of ECM components, especially if ascorbic acid is incorporated to the culture medium, as previously suggested (Grobe and Reichl, 2013). Long-term studies should be carried out on bioartificial corneas generated by tissue engineering to confirm or not this hypothesis. In addition, although most antibodies used in the present work were specific for human proteins, and despite our results demonstrated the presence of human antigens in the rabbit corneas, one cannot exclude the possibility that the ECM components found in the *in vivo* corneas may have been partially synthetized by the host cells. Furthermore, subsequent *in vivo* assays should also evaluate the role of the fibrin-agarose biomaterial by grafting the acellular scaffold in the rabbit cornea.

In summary, the results obtained in the present work are in agreement with previous reports suggesting that HWJSC may have partial epithelial differentiation potential *ex vivo* (Garzon et al., 2020), and that these cells could be used to generate HAC by tissue engineering. *In vivo* implantation of bioartificial corneas generated with this extra-corneal type of cells were able to generate a cornea properly integrated in the host. These corneas shared some phenotypic similarities with controls and with corneas generated with corneal epithelial cells, including a well-differentiated stroma and a basement membrane, suggesting that these bioengineered substitutes could have potential clinical usefulness. Compared with other types of extra-corneal sources of stem cells, HWJSC offer high including accessibility, proliferation and differentiation potential, and immune-privileged status (Borys-Wójcik et al., 2019). Future studies should be carried out to determine the therapeutic potential of HAC for the treatment of severe diseases affecting the corneal surface.

## Data Availability

The raw data supporting the conclusions of this article will be made available by the authors, without undue reservation.

## Supplementary material

SUPPLEMENTARY FIGURE S1 Immunofluorescence analysis of the OAC and HAC tissues kept *ex vivo* and grafted *in vivo* and control native human corneas (H) and rabbit corneas (R) using an anti-human mitochondria primary antibody. Scale bar: 50 µm (applicable to all images).
