# Supplementary material for: Development of stromal differentiation patterns in heterotypical models of artificial corneas generated by tissue engineering
Source: Front Bioeng Biotechnol. 2023 Mar 23;11:1124995. doi: 10.3389/fbioe.2023.1124995 (PMC10076743; doi:10.3389/fbioe.2023.1124995)
Supplement: Supplementary file 1 [file Table1.docx]

**Supplementary Table S1.** Quantitative analysis of the cornea extracellular matrix (ECM) components analyzed in the present work in orthotypical (OAC) and heterotypical (HAC) artificial corneas and controls (CTR). PSR: picrosirius red histochemistry for collagen fibers detection; VER: Verhoeff histochemistry for elastic fibers; RET: Gomori’s reticulin histochemistry for reticular fibers; AB: alcian blue histochemistry for proteoglycans; DCN: decorin immunohistochemistry; KER: keratocan immunohistochemistry; LUM: lumican immunohistochemistry; PAS: periodic acid-Schiff for glycosaminoglycans; NID: nidogen-1 immunohistochemistry. Averages and standard deviations are shown for each component and correspond to area fractions (for PSR, VER, RET, AB, DCN, KER and LUM) or intensity units (for PAS and NID). Controls correspond to native, normal human corneas (CTR).

|  | ***EX VIVO*** | | | | | | ***IN VIVO*** | | | | **CTR** |
| --- | --- | --- | --- | --- | --- | --- | --- | --- | --- | --- | --- |
|  | **7 Days** | | **14 Days** | | **21 Days** | | **3 Months** | | **12 Months** | |  |
|  | **OAC** | **HAC** | **OAC** | **HAC** | **OAC** | **HAC** | **OAC** | **HAC** | **OAC** | **HAC** |  |
| **PSR** | 0.23±0.32 | 0.26±0.38 | 0.18±0.31 | 0.26±0.38 | 0.25±0.4 | 0.12±0.28 | 31.75±11.28 | 40.91±12.32 | 43.79±8.1 | 58.50±6.46 | 41.47±10.99 |
| **VER** | 4.86±2.55 | 0.97±1.06 | 3.23±0.88 | 2.22±1.92 | 3.46±2.17 | 1.14±1.53 | 4.3±3.5 | 4.28±3.24 | 2.46±2.71 | 1.48±2.53 | 2.32±2.08 |
| **RET** | 3.54±3.78 | 1.45±1.58 | 1.83±2 | 2.09±2.06 | 3.12±2.63 | 1.66±1.76 | 3.97±4.29 | 3.27±3.6 | 2.72±2.9 | 2.55±2.77 | 1.84±2.17 |
| **AB** | 4.77±2.9 | 4.11±2.15 | 3.44±1.23 | 3.02±1.12 | 6.78±4.65 | 5.05±3.45 | 73.42±10.18 | 75.76±19.49 | 59.78±14.53 | 84.77±14.61 | 54.44±5.59 |
| **DCN** | 6.79±7.47 | 4.75±6.55 | 5.22±4.79 | 3.35±3.26 | 5.80±2.90 | 3.27±3.60 | 51.05±17.17 | 62.12±11.68 | 65.13±18.69 | 75.02±12.57 | 67.19±15.15 |
| **KER** | 54.26±11.48 | 37.75±4.02 | 40.83±8.05 | 42.57±1.13 | 43.62±5.29 | 36.74±6.39 | 90.95±10.66 | 115.86±8.84 | 117.85±3.11 | 112.59±12.54 | 147.03±22.79 |
| **LUM** | 2.57±7.09 | 0.17±0.09 | 1.66±1.24 | 1.17±0.83 | 1.92±0.91 | 0.61±0.75 | 10.86±10.41 | 9.45±9.02 | 7.07±7.57 | 8.61±7.43 | 13.19±3.96 |
| **PAS** | 39.13±9.11 | 53.25±30.78 | 41.38±4.87 | 47.88±30.87 | 52.63±11.5 | 54.63±36.77 | 135.75±35.15 | 123.88±53.3 | 116.5±47.03 | 125.75±61.44 | 157.88±22.92 |
| **NID** | 59.3±12.94 | 65.9±26.71 | 71±16.08 | 61.4±10.16 | 57.2±12.7 | 65.1±9.22 | 104.2±29.73 | 103.5±19.46 | 106.8±5.71 | 100.6±40.46 | 180±47.07 |

**Supplementary Table S2.** Statistical analysis of the quantitative results of the cornea extracellular matrix (ECM) components analyzed in the present work in orthotypical (OAC) and heterotypical (HAC) artificial corneas and controls (CTR). PSR: picrosirius red histochemistry for collagen fibers detection; VER: Verhoeff histochemistry for elastic fibers; RET: Gomori’s reticulin histochemistry for reticular fibers; AB: alcian blue histochemistry for proteoglycans; DCN: decorin immunohistochemistry; KER: keratocan immunohistochemistry; LUM: lumican immunohistochemistry; PAS: periodic acid-Schiff for glycosaminoglycans; NID: nidogen-1 immunohistochemistry. Statistical significance p values are shown for the comparison of specific groups of samples for each component. Statistically significant p values are highlighted with asterisks (*).

|  | **PSR** | **VERHOEFF** | **RET** | **AB** | **DCN** | **KER** | **LUM** | **PAS** | **NID** |
| --- | --- | --- | --- | --- | --- | --- | --- | --- | --- |
| **CTRvs. OAC 7 Days** | **0.0001*** | **0.0649** | **0.3822** | **0.0001*** | **0.0006*** | **0.0001*** | **0.0001*** | **0.0001*** | **0.0001*** |
| **CTRvs. OAC 14 Days** | **0.0001*** | **0.2786** | **0.9591** | **0.0001*** | **0.0006*** | **0.0001*** | **0.0001*** | **0.0001*** | **0.0001*** |
| **CTRvs. OAC 21 Days** | **0.0001*** | **0.1948** | **0.2344** | **0.0001*** | **0.0006*** | **0.0001*** | **0.0001*** | **0.0001*** | **0.0001*** |
| **CTRvs. OAC 3 Months** | **0.0649** | **0.1605** | **0.9591** | **0.0029** | **0.0812** | **0.0001*** | **0.4418** | **0.1605** | **0.0001*** |
| **CTRvs. OAC 12 Months** | **0.7984** | **0.9591** | **0.7209** | **0.6453** | **0.9497** | **0.0006*** | **0.0649** | **0.0649** | **0.0001*** |
| **CTRvs. HAC 7 Days** | **0.0001*** | **0.5737** | **0.8784** | **0.0001*** | **0.0006*** | **0.0001*** | **0.0001*** | **0.0001*** | **0.0001*** |
| **CTRvs. HAC 14 Days** | **0.0001*** | **0.5053** | **0.2344** | **0.0001*** | **0.0006*** | **0.0001*** | **0.0001*** | **0.0001*** | **0.0001*** |
| **CTRvs. HAC 21 Days** | **0.0001*** | **0.7984** | **0.5053** | **0.0001*** | **0.0006*** | **0.0001*** | **0.0001*** | **0.0001*** | **0.0001*** |
| **CTRvs. HAC 3 Months** | **0.7984** | **0.1303** | **0.8784** | **0.0205** | **0.6991** | **0.0069** | **0.2786** | **0.0829** | **0.0001*** |
| **CTRvs. HAC 12 Months** | **0.001*** | **0.6453** | **0.7209** | **0.0006*** | **0.3449** | **0.0046** | **0.2344** | **0.1048** | **0.0001*** |
| **OAC 7 Days vs. OAC 14 Days** | **0.8784** | **0.2344** | **0.2344** | **0.4418** | **0.5053** | **0.0281** | **0.0104** | **0.5737** | **0.0147** |
| **OAC 14 Days vs. OAC 21 Days** | **0.8784** | **0.5053** | **0.0829** | **0.1948** | **0.8784** | **0.5737** | **0.7209** | **0.0649** | **0.0029** |
| **OAC 21 Days vs. OAC 3 Months** | **0.0001*** | **0.7984** | **0.9591** | **0.0003*** | **0.0006*** | **0.0001*** | **0.0069** | **0.0001*** | **0.0001*** |
| **OAC 3 Months vs. OAC 12 Months** | **0.0379** | **0.1303** | **0.6453** | **0.0649** | **0.1605** | **0.0001*** | **0.4418** | **0.1048** | **0.0001*** |
| **HAC 7 Days vs. HAC 14 Days** | **0.9591** | **0.1303** | **0.1303** | **0.3282** | **0.9591** | **0.0104** | **0.0001*** | **0.3822** | **0.0206** |
| **HAC 14 Days vs. HAC 21 Days** | **0.7984** | **0.1605** | **0.5053** | **0.1303** | **0.7984** | **0.0006*** | **0.0649** | **0.6453** | **0.5053** |
| **HAC 21 Days vs. HAC 3 Months** | **0.0001*** | **0.0206** | **0.9591** | **0.0003*** | **0.0006*** | **0.0001*** | **0.0029** | **0.0069** | **0.0003*** |
| **HAC 3 Months vs. HAC 12 Months** | **0.0069** | **0.0147** | **0.8784** | **0.6943** | **0.0426** | **0.4418** | **0.9591** | **0.6453** | **0.0046** |
| **OAC 7 Days vs. HAC 7 Days** | **0.8784** | **0.0029** | **0.3822** | **0.6453** | **0.7984** | **0.0206** | **0.2344** | **0.4418** | **0.0147** |
| **OAC 14 Days vs. HAC 14 Days** | **0.7984** | **0.5053** | **0.2344** | **0.5053** | **0.3282** | **0.7209** | **0.5737** | **0.4418** | **0.0206** |
| **OAC 21 Days vs. HAC 21 Days** | **0.7209** | **0.0069** | **0.4418** | **0.5053** | **0.0649** | **0.0147** | **0.0104** | **0.5737** | **0.0498** |
| **OAC 3 Months vs. HAC 3 Months** | **0.3282** | **0.9591** | **0.4418** | **0.1892** | **0.2823** | **0.001*** | **0.5737** | **0.1048** | **0.4418** |
| **OAC 12 Months vs. HAC 12 Months** | **0.0006*** | **0.9591** | **0.9591** | **0.0069** | **0.3282** | **0.1048** | **0.7209** | **0.5053** | **0.0001*** |
