# Supplementary figures and images for: Development of stromal differentiation patterns in heterotypical models of artificial corneas generated by tissue engineering
Source: Front Bioeng Biotechnol. 2023 Mar 23;11:1124995. doi: 10.3389/fbioe.2023.1124995 (PMC10076743; doi:10.3389/fbioe.2023.1124995)

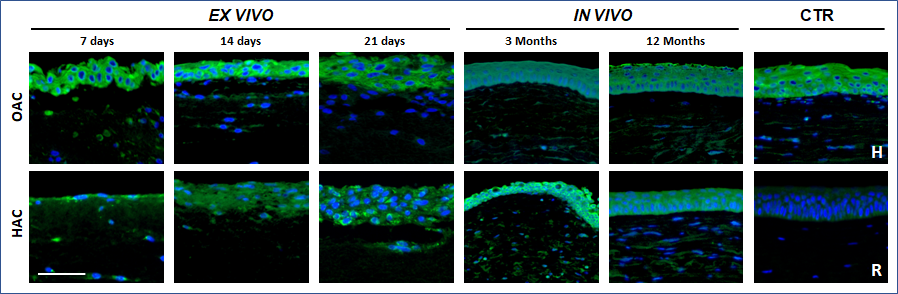

Supplement: Supplementary file 2 [file Image1.TIF]
